# Study on the Alkali-Activated Mechanism of Yellow River Sediment-Based Ecological Cementitious Materials

**DOI:** 10.3390/ma18071559

**Published:** 2025-03-29

**Authors:** Ge Zhang, Enhui Jiang, Kunpeng Li, Huawei Shi, Chen Chen, Chengfang Yuan

**Affiliations:** 1 Yellow River Institute of Hydraulic Research, Yellow River Water Conservancy Commission, Zhengzhou 450003, China; gezhangyrihr@163.com (G.Z.); likunpeng@hky.yrcc.gov.cn (K.L.); 15538352232@163.com (H.S.); 15617633649@163.com (C.C.); 2 Key Laboratory of Lower Yellow River Channel and Estuary Regulation, Ministry of Water Resources, Zhengzhou 450003, China; 3 Yellow River Laboratory, Zhengzhou 450003, China; 4 College of Civil Engineering, Zhengzhou University, Zhengzhou 450001, China

**Keywords:** Yellow River sediment, alkali-activation, strength, characteristic products, microstructure

## Abstract

As one of the key components in geopolymer systems, the activator significantly influences the properties of cementitious materials. This study investigates the effects of key activator parameters, specifically alkali equivalent and activator modulus, on the setting time, workability, hydration characteristics, compressive strength, and splitting tensile strength of Yellow River sediment-based slag eco-friendly cementitious materials. Tests such as setting time, slump, flowability, hydration heat, and strength were conducted to evaluate these effects. Additionally, X-ray diffraction (XRD), differential thermal analysis (DTA), mercury intrusion porosimetry (MIP), and scanning electron microscopy with energy dispersive spectroscopy (SEM-EDS) tests were conducted to investigate the mechanisms and variations in microstructural properties. The results indicate that the alkali equivalent and activator modulus significantly affect the setting time, workability, reaction process, and strength of Yellow River sediment-based eco-friendly cementitious materials. An excessively high or low alkali equivalent and activator modulus result in either insufficient or excessive activation, adversely affecting the densification process of the hardened matrix. When the alkali equivalent is 5% and the activator modulus is 1.2, the matrix demonstrates superior flowability, well-regulated and sustained heat evolution during hydration, and achieves compressive and splitting tensile strengths of 61.68 MPa and 4.37 MPa, respectively. Under optimal alkaline conditions, slag dissolution, hydrolysis of silicon–oxygen and aluminum–oxygen tetrahedra, and the formation of low-calcium calcium silicate hydrate (C-S-H) and calcium aluminum silicate hydrate (C-A-S-H) phases are effectively promoted, leading to the development of a wrinkled three-dimensional polymeric gel structure. This structure fills the matrix pores, optimizes the pore structure, and contributes to strength development.

## 1. Introduction

**Figure 1:**
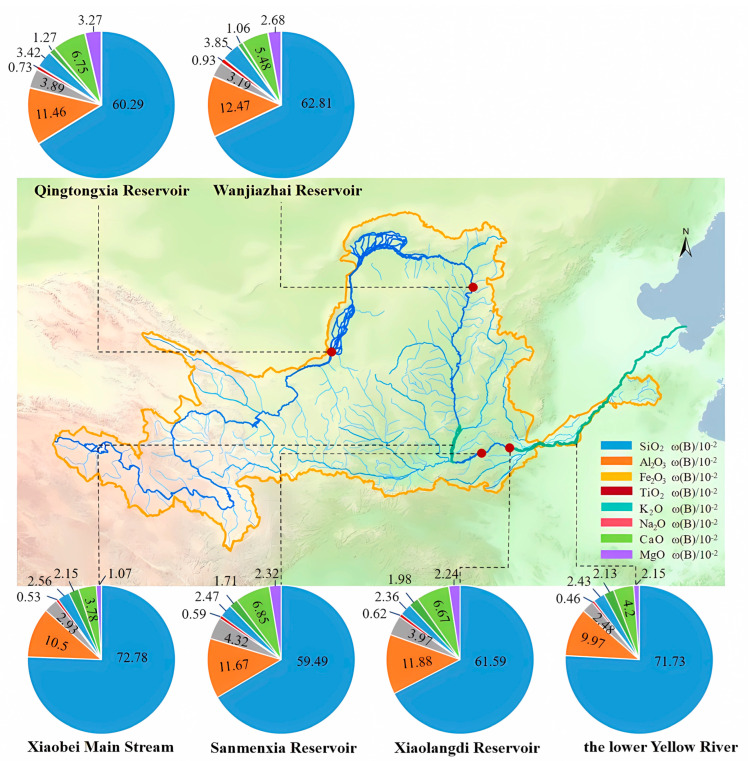
Properties of sediment resources in the Yellow River.

The protection and sustainable management of the Yellow River is a long-term strategic priority crucial to the socioeconomic development and ecological stability of China. The Yellow River Basin faces severe sedimentation challenges due to factors such as low water discharge, excessive sediment load, and an imbalanced water–sediment relationship. Sediment deposition is a persistent technical issue affecting flood control measures, reservoir storage capacity, and the operational efficiency of irrigation systems. Meanwhile, the demand for construction aggregates in China is enormous, with approximately 20 billion tons of sand and gravel consumed annually [1]. The depletion of natural sand resources has necessitated the exploration of alternative materials with stable supply potential [2,3]. In this context, Yellow River sediment has emerged as a promising substitute due to its abundance and economic viability. The primary constituent of Yellow River sediment is silicon dioxide (SiO_2_) (Figure 1), which makes it a viable alternative for cementitious applications. Utilizing Yellow River sediment in construction materials offers substantial environmental and economic benefits.

Ordinary Portland Cement (OPC) remains the dominant cementitious material in the construction sector and is pivotal in global infrastructure development. However, OPC production is highly energy-intensive and contributes significantly to carbon emissions and environmental degradation [4]. To mitigate these adverse effects, there is an urgent need to develop sustainable, low-carbon, and high-performance cementitious materials. Alkali-activated materials (AAMs) have emerged as a viable alternative, produced through the chemical activation of aluminosilicate sources using alkaline activators [5,6]. These materials exhibit superior mechanical performance [7,8,9], excellent resistance to high temperatures [10,11,12], corrosion resistance [13,14], and acid resistance [15,16] while also offering advantages such as lower energy consumption (no high-temperature calcination required), reduced CO_2_ emissions, and the ability to incorporate industrial by-product [17]. Consequently, researchers have explored alkali-activation techniques to incorporate solid waste and Yellow River sediment into eco-friendly cementitious materials.

Several studies have demonstrated the feasibility of utilizing Yellow River sediment in alkali-activated cementitious systems. Wang et al. used Yellow River silt as a raw material combined with blast furnace slag to prepare flood-prevention stone. The study showed that when Ca(OH)_2_ (5 wt.%) and NaOH (0.5 wt.%) as compound alkali activators are added into the mixture, the strength (12.3 MPa at 28 days) of the specimen can also meet requirements [18]. He et al. studied the combination of Yellow River silt and red mud for the preparation of sintered bricks. Experimental evidence revealed that when the RM content was 40% by mass ratio, the sintering temperature was 1050 °C, the sintering time was 2 h, and the composition demonstrated optimal performance, attaining a compressive strength of 39.1 MPa [19]. Raza et al. evaluated Yellow River sand (YRS) as a sustainable alternative to quartz sand in engineered cementitious composites (ECCs) [20]. Their study showed that 25% YRS replacement improves ECC durability and mechanical performance under freeze–thaw conditions.

Sheng et al. utilized alkali-activated Yellow River sediment to prepare foam concrete [21]. The study demonstrated that as the content of Yellow River sediment increased, the compressive strength of the foam concrete progressively decreased, especially in the early stages (3 and 7 days), where the effect was more noticeable. When the Yellow River sediment content exceeded 15%, a significant reduction in compressive strength was observed. In a related study, Jin et al. prepared alkali-activated cementitious materials using red mud and Yellow River sand and investigated their mechanical properties [22]. Yuan et al. investigated Yellow River sand (YRS) in engineered cementitious composites (ECCs), finding that 100% YRS substitution enhances strength, while 75% substitution provides optimal ductility, promoting sustainable concrete applications [23].

The study found that when 40% Yellow River sand was incorporated, the compressive strength of the geopolymer cementitious material reached 48.8 MPa at 28 days, which is comparable to that of the material without Yellow River sand. Li et al. used alkali-activated Yellow River sediment to prepare artificial flood-prevention stones [24,25]. By employing a pressing molding method and incorporating 67.3% Yellow River sediment along with a composite activation method using 5% Ca(OH)_2_, 0.2% NaOH, and 7.5% CaSO_4_, the maximum strength of the composite material reached 14.4 MPa after 90 days of curing. Jing et al. found that increasing the content of Ca(OH)_2_ could enhance the compressive strength of the samples [26]. When the Ca(OH)_2_ content was 12.5%, and 77.5% Yellow River sediment was incorporated, the 90-day compressive strength of the artificial flood-prevention stones reached a maximum value of 6.9 MPa. Li et al. prepared composite cementitious materials by alkali-activating Pisha sandstone (one of the main sources of Yellow River sediment) [27]. The study demonstrated that curing duration, activator composition, and the proportion of mineral admixtures play a crucial role in influencing the performance of composite cementitious materials. The optimal mix design was achieved with 74% Pisha sandstone and 13.8% fly ash, yielding the highest compressive strength of 20.3 MPa.

In summary, alkali activators have a significant impact on the performance of cementitious materials. From the perspective of the activator system composition, most existing studies focus on the formulation of activators, synthesis processes, and the effects of specific types of activators on the matrix. Scholars have proposed various optimal mix proportions for different types of raw materials, with significant discrepancies in technical parameters, such as water glass modulus and alkali dosage [28,29]. Even for the same type of raw material, the optimal parameters may vary considerably [30]. The primary reason lies in the fact that even for identical materials, differences in origin lead to variations in chemical composition and combinations, making it impossible to establish a unified standard.

Yellow River sediment exhibits notable differences from conventional construction sand in terms of particle-size distribution and physicochemical characteristics. Its particles are predominantly fine, mainly within the silt and clay particle range (<0.075 mm), with flaky or angular shapes, rough surfaces, large specific surface areas, and high clay-mineral contents. These characteristics result in distinct performance differences when used as aggregate in alkali-activated materials compared to those using traditional river sand. Therefore, existing research findings cannot be directly applied to predict the performance of alkali-activated Yellow River sediment eco-cementitious materials. It is necessary to establish a dedicated parameter optimization system specifically tailored to the unique properties of Yellow River sediment.

From the perspective of performance indicators, the research mainly emphasizes compressive strength, which is generally low, while studies on tensile strength and flexural strength are relatively limited. Additionally, issues of insufficient activation and over-activation persist, both of which hinder long-term strength development. The reaction mechanisms of alkali-activated cementitious materials are still not fully understood, and there is a lack of effective methods for controlling alkali activation, making it challenging to systematically guide engineering applications.

Based on this background, this study investigates the performance and mechanism of alkali-activated Yellow River sediment (YRS) cementitious materials. The research analyzes the effects of different alkali equivalents and activator moduli on the setting time, workability, reaction process, and strength of YRS-based eco-friendly cementitious materials. Microscopic testing methods, including DTA, XRD, SEM-EDS, and MIP, are used to examine characteristic products, pore structure, and matrix microstructure. The study aims to explain the alkali-activation regulation mechanism and strength development mechanism of YRS-based eco-friendly cementitious materials, providing a reference for their research and application.

## 2. Materials and Methods

### 2.1. Raw Material and Mixed Proportion

#### 2.1.1. Raw Material

**Figure 2:**
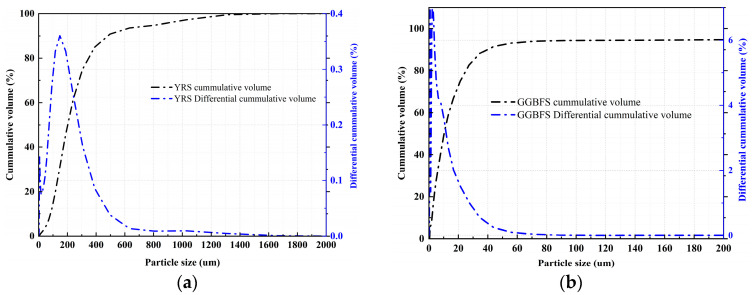
Particle-size distribution curves of YRS and GGBS. (**a**) YRS, (**b**) GGBFS.

The raw materials used in the experiment mainly consist of YRS, slag, NaOH, and water glass. The YRS was collected from the Xixiayuan Reservoir in Henan Province in a moist state and was subsequently dried before being used as a raw material. The chemical compositions and particle sizes of the YRS and slag were analyzed using XRF (Shimadzu Corporation, Kyoto, Japan) and a Malvern Mastersizer-2000 laser particle-size analyzer (Malvern Panalytical, Worcestershire, UK). The results are presented in Table 1 and Figure 2, respectively. The slag used in this study is of grade S105, with a median particle size of 10.28 μm, a hydraulic coefficient of 2.17, an activity coefficient of 0.48, and an alkaline coefficient of 1.14. Water glass, commonly known as sodium silicate, is a general term for Na_2_O·*n*SiO_2_, where *n* is typically referred to as the modulus. It is a transparent, glassy solution composed of alkali metal silicates. The chemical composition of water glass primarily depends on the molecular ratio *n* between SiO_2_ and alkali metal oxides (Na_2_O or K_2_O), known as the water glass modulus. For this experiment, liquid sodium silicate produced by Shandong Yourui Chemical Co., Ltd. (Heze, China). was used, with the physicochemical parameters provided in Table 2. Pure NaOH was used to adjust the water glass to the desired modulus, and the modulus adjustment equation is shown in Formula (1). Tap water was used for mixing throughout the experiment.

(1) $$Na_{2}O\cdot 2.18SiO_{2}+NaOH\to Na_{2}O\cdot nSiO_{2}+H_{2}O$$

#### 2.1.2. Mixed Proportion

Alkali equivalent refers to the mass percentage of Na_2_O in the alkali activator relative to the raw mineral powder (wt.%), while activator modulus represents the molar ratio of SiO_2_ to Na_2_O in the activator. These two parameters collectively govern the core alkali-activated reaction process of ‘aluminosilicate dissolution–polycondensation’, wherein the alkali equivalent controls the dissolution rate of aluminosilicate precursors, and the activator modulus regulates the polycondensation behavior of dissolved species.

Table 3 shows the mix proportion of YRS ecological cementitious materials. Before mixing, the pre-weighed NaOH was fully dissolved in sodium silicate. The materials were mixed using a single horizontal shaft concrete mixer. First, YRS and ground granulated blast furnace slag (GGBS) were dry-mixed for 180 s, followed by the addition of water and further mixing for another 180 s. Then, sodium silicate was introduced and blended for 120 s. The fresh mix was cast into molds and compacted on a vibration table for 60–90 s. After 24 h of curing at room temperature, the specimens were demolded and stored in a curing chamber at 20 ± 2 °C and 95 ± 5% relative humidity until the 28-day age [30,31,32].

### 2.2. Experimental Method

In this experiment, the effects of alkali equivalent and modulus on the workability, mechanical properties, reaction progress, characteristic products, and microstructural properties of YRS ecological cementitious materials were analyzed using various tests. These tests included setting time, compressive and splitting tensile strength, hydration heat, TGA, XRD MIP, and SEM. Table 4 shows the grouping of the test, including the size and number of each test and specimen.

#### 2.2.1. Setting Time Test

Due to the fact that a high dosage of alkali can significantly accelerate the setting of a paste, conventional methods for measuring the setting time of cement are not suitable for alkali-activated pastes. The setting time was tested according to GB/T 35159 (flash setting admixtures for shotcrete) [33]. The water–cement ratio used for determining the setting time of the cement paste was 0.35, and the average value was taken from three experiments in each group.

#### 2.2.2. Workability Test

The workability of the mixture was tested using a micro-collapse cylinder with an upper mouth of 50 mm, a lower mouth of 100 mm, a height of 150 mm, and a 60 cm × 60 cm plate [34].

#### 2.2.3. Hydration Heat Test

The hydration heat was measured using a TAM AIR isothermal calorimeter (TA Instruments, New Castle, DE, USA). The test was conducted at a temperature of 25 °C, with a temperature fluctuation range of less than 0.02 °C and a measurement accuracy of ±20 μW. The duration of the test was 4 days.

#### 2.2.4. Strength Test

Compressive strength and splitting tensile strength tests were conducted using 100 mm × 100 mm × 100 mm cubic specimens, following the requirements outlined in GB/T 50081-2019 “Standard for Test Methods of Physical and Mechanical Properties of Concrete” [35].

#### 2.2.5. X-Ray Diffraction Analysis

The phase composition of the specimens was analyzed using a Japanese Physical X-ray diffractometer (Shimadzu Corporation, Kyoto, Japan). After drying and grinding, the sample was placed in a glass sample holder for testing. The analysis was conducted with a sampling interval of 0.04° (2θ) and a scanning speed of 2°/min. The diffraction data were collected within a scanning range of 5°–70° (2θ) to identify the crystalline phases present in the cementitious matrix.

#### 2.2.6. Thermogravimetric Analysis

A ZCT-B simultaneous thermal analyzer (Beijing Jingyi Hi-Tech Instrument Co., Ltd., Beijing, China) was employed, with the test sample weighing approximately 15 mg. The heating rate was controlled at 10 °C/minute, and the maximum temperature was raised to 1000 °C. The DTA curves for each group of samples were obtained.

#### 2.2.7. Porosity Test

The porosity and pore-size distribution of the hardened paste were analyzed using the mercury intrusion method. Small-size paste specimens were prepared for standard curing (20 ± 2 °C, RH > 95%). After curing for 28 days, the hydrated samples were broken using pliers for testing. The porosity was measured using a Poremaster-33T automatic mercury porosimeter (Anton Paar Quanta Tec Inc., Boynton Beach, FL, USA) with an aperture measurement range of 3.5 nm to 360,000 nm [36,37].

#### 2.2.8. Scanning Electron Microscopy Test

The microstructure of the cement paste samples was observed using a Sigma 300 field emission environmental scanning electron microscope (Carl Zeiss AG, Oberkochen, Germany). After curing to the 28d age, the samples were broken with pliers, and hydration was stopped before testing [38].

## 3. Experiment Results and Analysis

### 3.1. Effect of Alkali Activator Characteristic Parameters on Setting Time

**Figure 3:**
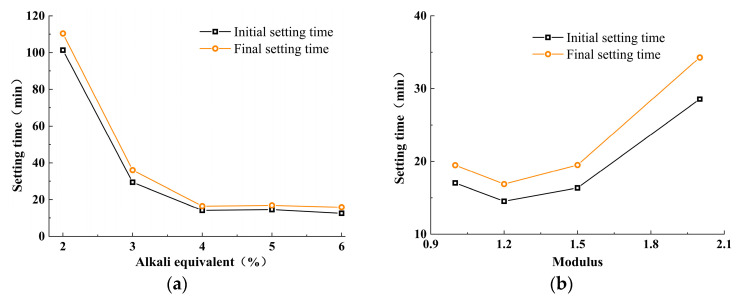
Effect of alkali activator characteristic parameters on setting time for (**a**) alkali equivalent and (**b**) modulus.

Figure 3 shows the effect of alkali activator characteristic parameters on the setting time of the paste. As shown in Figure 3, both modulus and alkali equivalent demonstrate a two-stage change, however, the influence is different.

Figure 3a illustrates the effect of alkali equivalent content on the setting time of the paste. When the alkali equivalent is less than 4%, the setting time decreases rapidly as the alkali equivalent increases. Specifically, when the alkali equivalent reaches 4%, the initial and final setting times decrease significantly to 14.1 min and 16.5 min, respectively. Beyond this point, when the alkali equivalent exceeds 4%, the setting time changes only slightly with further increases in the alkali equivalent. At an alkali equivalent of 6%, the initial and final setting times are 12.5 min and 15.9 min, respectively. Overall, the setting time of the eco-friendly cementitious material made from YRS increases as the activator modulus rises. This is because activators with higher moduli have greater viscosity, which reduces the migration rate of alkali metal cations and weakens the interactions between reactive substances, thereby slowing down the polymerization reaction rate. This leads to an increase in the concentration of high-polymer silicates and a decrease in the activation sites of siloxane groups, delaying the setting and hardening process.

Figure 3b illustrates the effect of modulus on the setting time of the paste. It can be observed that as the modulus of the activator increases, the setting time follows a two-stage pattern: it first decreases and then increases, reaching a minimum value at a modulus of 1.2. At this point, the initial and final setting times are 14.5 min and 16.9 min, respectively. However, further increasing the modulus leads to an extension of the setting time. When the modulus reaches 2.0, the initial and final setting times increase to 28.6 min and 34.3 min, respectively. This behavior is attributed to the fact that as the modulus increases, the reaction rate of small-molecule silicates (monosilicates, chain, and cyclic trimers) decreases [39,40]. The concentration of high-polymer silicates increases, and the activation sites of siloxane groups decrease, thereby delaying the setting and hardening process. Overall, both the modulus of the alkali activator and the alkali content have a significant impact on the setting time, with the setting time generally increasing as the activator modulus increases and the alkali equivalent decreases.

### 3.2. Effect of Alkali Activator Characteristic Parameters on Workability

**Figure 4:**
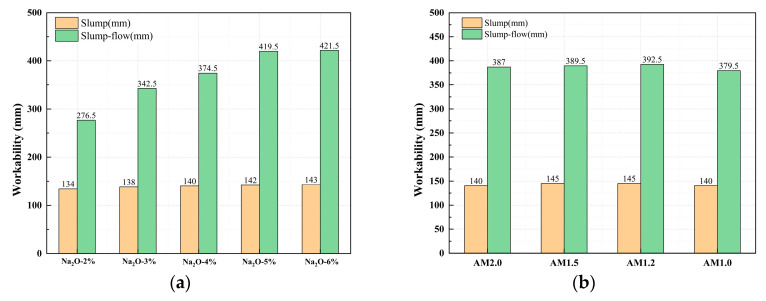
Effect of alkali activator characteristic parameters on workability for (**a**) alkali equivalent and (**b**) modulus.

Figure 4 illustrates the influence of the alkali equivalent and activator modulus on the workability of the mixture. From Figure 4a, it can be observed that as the alkali equivalent increases, both the slump and spread of the mixture show an increasing trend. For mixtures with 2% to 6% Na_2_O, the slump values are 134 mm, 138 mm, 140 mm, 142 mm, and 143 mm, respectively. Compared to the 2% Na_2_O mixture, the slump of the 3% to 6% Na_2_O mixtures increases by 2.99%, 4.48%, 5.97%, and 6.72%, respectively. The spread values for the 2% to 6% Na_2_O mixtures are 276.5 mm, 342.5 mm, 374.5 mm, 419.5 mm, and 421.5 mm, respectively. Compared to the 2% Na_2_O mixture, the spread of the 3% to 6% Na_2_O mixtures increases by 23.87%, 35.44%, 51.72%, and 52.44%, respectively. When the alkali content exceeds 5%, the workability of the mixtures shows little difference.

Overall, increasing the alkali content generally improves the workability of the concrete, leading to a higher slump and spread. This is because a higher alkali equivalent accelerates the dissolution of raw materials, which reduces the cohesion of the mixture paste and facilitates better flow. As a result, the initial fluidity improves, and the slump becomes larger. However, the slump may decrease rapidly due to the fast reaction. On the other hand, mixtures with lower alkali content react more slowly, resulting in a smaller initial slump but longer fluidity retention. Therefore, compared to slump, increasing the alkali equivalent has a more significant effect on enhancing the spread of the mixture.

From Figure 4b, it can be seen that when the alkali equivalent is fixed, the slump values for the AM2.0 to AM1.0 specimens are 140 mm, 145 mm, 145 mm, and 140 mm, respectively, and the spread values are 387 mm, 389.5 mm, 392.5 mm, and 379.5 mm, respectively. Overall, the workability of mixtures with different moduli is good, with little difference in slump and spread. Comparatively, the AM1.2 mixture exhibits the best flow performance. In comparison to the activator modulus, the alkali equivalent has a more significant impact on the workability of the mixture.

### 3.3. Effect of Alkali Activator Characteristic Parameters on the Early Reaction Process

Compared to traditional cement-based materials, alkali-activated materials exhibit more intense early stage reactions, primarily manifested in their rapid dissolution–polymerization process and significant exothermic behavior. Through isothermal calorimetry testing, we can quantitatively analyze the variation patterns of key parameters such as heat release rate, cumulative heat release, and exothermic peak time to characterize their thermal features. This approach enables an in-depth investigation into the influence mechanisms of alkali equivalent (Na_2_O content) and activator modulus (SiO_2_/Na_2_O molar ratio) on the early stage hydration process of the matrix.

**Figure 5:**
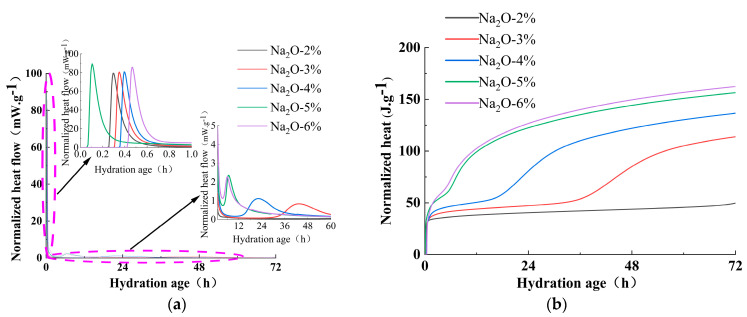
Effect of alkali equivalent on heat release curves for (**a**) heat evolution rate and (**b**) accumulated hydration heat.

Figure 5 illustrates the effect of the alkali equivalent on the hydration heat release rate and cumulative hydration heat of the mixture. The hydration heat release process of alkali-activated cementitious materials can be divided into five stages: initial reaction period, induction period, acceleration period, deceleration period, and stabilization period. The hydration heat release curve of alkali-slag cement typically exhibits one main initial peak and an additional initial peak that occurs before the induction period, followed by an acceleration peak after the induction period. The main initial peak and the additional initial peak occur very closely in time and merge into a single peak. The initial heat release peak is due to the wetting and dissolution of slag particles, while the additional initial peak arises from the reaction between Ca^2+^ ions dissolved from slag particles and the anions or anionic groups dissolved from the water glass. This reaction and its products (C-A-S-H) have a significant impact on the setting time and strength of the cement paste. The precipitation of a large amount of C-A-S-H gel leads to the appearance of the induction period, and the acceleration peak can be attributed to the acceleration of the slag reaction.

From Figure 5a, it can be observed that as the alkali equivalent increases, the first peak occurs earlier and becomes higher, the acceleration peak also becomes higher and occurs earlier, the induction period shortens, and the cumulative hydration heat increases. This is mainly because increasing the amount of alkali components enhances the alkalinity in the alkali-slag cement system, leading to an increase in [SiO_4_]^4−^. This accelerates the dissolution and disintegration of slag, speeds up the reaction between Ca^2+^ and [SiO_4_]^4−^, and promotes an increase in the reaction rate of alkali-slag cement [41]. As a result, the heat release peak occurs earlier, and the total hydration heat increases.

As observed in Figure 5b, an increase in alkali equivalent notably enhances the hydration heat release of the mixture. After 24 h, the cumulative heat releases for mixtures with Na_2_O contents of 2%, 3%, 4%, 5%, and 6% are recorded as 40.4 J/g, 47.2 J/g, 80.8 J/g, 122.5 J/g, and 126.8 J/g, respectively, demonstrating a progressive rise in exothermic activity with higher alkali dosages. The hydration heat release increases with the increase in alkali equivalent. After 72 h of cumulative heat release, the hydration heat releases for 2% to 6% Na_2_O mixtures are 49.8 J/g, 113.9 J/g, 136.6 J/g, 156.4 J/g, and 162.4 J/g, respectively. Overall, the hydration heat release rate is the fastest for the 3% Na_2_O mixture, but the total hydration heat release is still lower than that of high-alkali pastes. The hydration heat releases for 4% to 6% Na_2_O pastes are 19.95%, 37.36%, and 42.59% higher than that of the 3% Na_2_O paste, respectively. When the Na_2_O content exceeds 5%, the change in hydration heat release is minimal, which aligns with the strength-development pattern.

**Figure 6:**
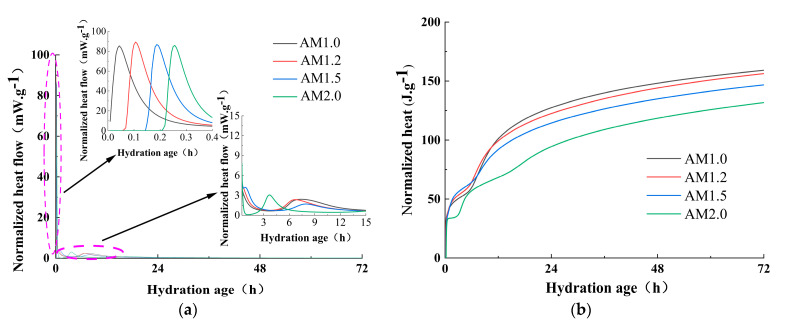
Effect of alkali activator modulus on heat release curves for (**a**) heat evolution rate and (**b**) accumulated hydration heat.

Figure 6 illustrates the influence of the activator modulus on the hydration heat release rate and the cumulative hydration heat of the mixture. Since all systems fall within the alkali-silicate-activated slag cementitious material category, the hydration heat release curves for varying activator moduli exhibit similar trends to those observed for different alkali equivalents. From Figure 6a, it can be observed that as the activator modulus decreases, the first peak occurs earlier and becomes higher, the acceleration peak also becomes higher and occurs earlier, the induction period shortens, and the cumulative hydration heat increases. This is mainly because, as the modulus decreases, the reaction rate of small-molecule silicates (monosilicates, chain and cyclic trimers) increases, and the activation sites of siloxane groups increase. This accelerates the dissolution and disintegration of slag, speeds up the reaction between Ca^2+^ and [SiO_4_]^4−^, and promotes an increase in the reaction rate of alkali-slag cement. As a result, the heat release peak occurs earlier, and the total hydration heat increases.

Figure 6b shows that reducing the activator modulus significantly increases the hydration heat release of the mixture. After 24 h, the cumulative heat release for AM2.0 to AM1.0 mixtures is 94.5 J/g, 114.5 J/g, 122.5 J/g, and 127.5 J/g, respectively, indicating a progressive increase in hydration heat release as the activator modulus decreases. After 72 h, the cumulative hydration heat releases for AM2.0 to AM1.0 mixtures are 131.8 J/g, 146.9 J/g, 156.4 J/g, and 159.2 J/g, respectively. While the AM2.0 mixture exhibits the fastest hydration heat release rate, its total cumulative hydration heat release is lower than that of mixtures with lower activator moduli. The hydration heat releases for AM1.0 to AM1.5 mixtures are 20.74%, 18.65%, and 11.40% higher than that of the AM2.0 mixture, respectively. When the activator modulus is below 1.2, the change in hydration heat release is minimal, which aligns with the strength-development pattern.

Overall, both the alkali equivalent and the water glass modulus affect the hydration heat release rate and cumulative hydration heat of the mixed system. In comparison, the effect of the alkali equivalent is more significant.

### 3.4. Effect of Alkali Activator Characteristic Parameters on Strength

The alkali equivalent, which is the mass percentage of Na_2_O in the alkali activator relative to the raw material slag powder, is a key factor influencing the degree of alkali-activation progress [42]. Its dosage directly affects the dissolution–depolymerization rate of silicon and aluminum in the slag powder [43].

**Figure 7:**
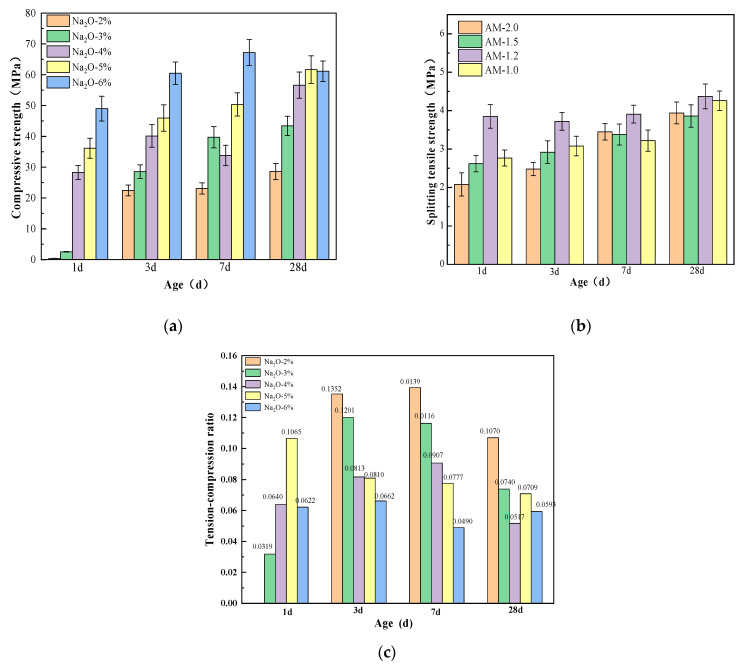
Effects of alkali equivalent on strength for (**a**) compressive strength, (**b**) splitting tensile strength, and (**c**) tension–compression ratio.

Figure 7 shows the effect of the alkali equivalent on the strength of the paste. From Figure 7, it can be observed that the alkali equivalent has a significant impact on the strength development of alkali-activated slag cementitious materials. At 1 day, the compressive strengths of specimens with 2% to 6% Na_2_O are 0.41 MPa, 2.54 MPa, 28.31 MPa, 36.15 MPa, and 49.04 MPa, respectively. The splitting tensile strength of the 2% Na_2_O specimen is too low to be measured, while the splitting tensile strengths of the 3% to 6% Na_2_O specimens are 0.81 MPa, 1.81 MPa, 3.85 MPa, and 3.05 MPa, respectively. Increasing the alkali equivalent significantly accelerates the early strength development of the specimens. In particular, when the alkali equivalent exceeds 4%, the 1 day splitting tensile strength can reach 62% to 88% of the 28 day strength, and the 1 day compressive strength can reach 50% to 80% of the 28 day strength. The higher the alkali equivalent, the more pronounced the strength improvement.

As the curing age increases, the compressive and splitting tensile strengths of specimens with different mix ratios exhibit varying trends. Both excessively low and high alkali equivalents hinder strength development, leading to weak strength growth or even a decline in later stages. The optimal improvement in compressive and splitting tensile strengths is observed at 5% Na_2_O. When the alkali content is too low, the dissolution rates of Al and Si are insufficient, resulting in incomplete polymerization and a loose, porous internal structure that negatively impacts strength development. Conversely, when the alkali content is too high, the reaction proceeds too rapidly, forming hydration products on the surface of slag particles that create a protective film [44,45], which limits further reaction and strength gain [46]. Additionally, excess Na_2_SiO_3_ is detrimental to the stability of the cementitious material system, leading to slow strength development in the later stages [47].

Overall, the mechanical properties of eco-friendly cementitious materials made from YRS show a trend of first increasing and then decreasing with an increase in alkali equivalent [48]. Within a certain range, increasing the alkali equivalent can better dissolve silicon–aluminum raw materials, fully activating the potential reactivity of slag [49,50]. However, an excessively high alkali equivalent leads to an overabundance of OH− in the system and premature deposition of silicate–aluminate products [44], hindering the normal progress of the polymerization reaction [51]. At the same time, the presence of too many alkali metal ions (M+) in the structure affects the electronegativity of the structure, impeding its formation.

**Figure 8:**
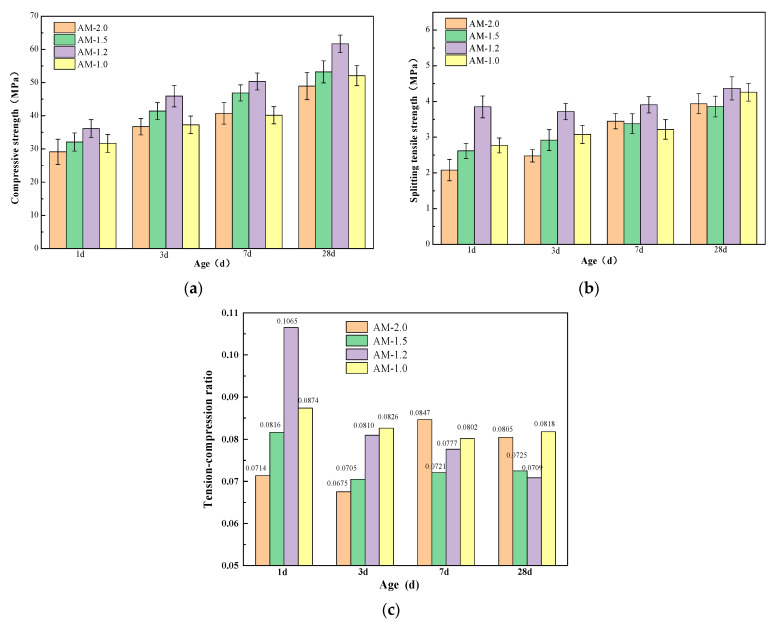
Effects of alkali activator modulus on strength for (**a**) compressive strength, (**b**) splitting tensile strength, and (**c**) tension–compression ratio.

The modulus of water glass is a critical parameter influencing the performance of alkali-activated cementitious materials. Figure 8 illustrates the effect of alkali activator modulus on the strength of the paste. From Figure 8, it can be observed that as the activator modulus decreases, the compressive and splitting tensile strengths of the eco-friendly cementitious materials made from YRS at different curing ages initially increase and then decrease. This trend suggests that there is an optimal range for the activator modulus, where the material exhibits the best strength development, and deviations from this range lead to reduced strength.

From Figure 8, it can be observed that, under a fixed alkali equivalent, the compressive and splitting tensile strengths of specimens with different activator moduli increase rapidly with the increase in curing age, exhibiting significant early strength characteristics. After 1 day, the compressive strength of AM2.0~1.0 specimens can reach about 60% of the 28 day strength. The splitting tensile strength of AM2.0~1.0 specimens after 1 day can reach about 53~88% of the 28 day strength, with the AM1.2 specimen showing the highest splitting tensile strength at 1 day, significantly higher than other mix-ratio specimens. From the figure, it can also be seen that the effect of different activator moduli on strength is quite noticeable. At 1 day, the compressive strengths of AM2.0~1.0 specimens are 29.15 MPa, 32.10 MPa, 36.15 MPa, and 31.7 MPa, respectively, while the splitting tensile strengths are 2.08 MPa, 2.62 MPa, 3.85 MPa, and 2.77 MPa, respectively. The AM1.2 specimen exhibits the highest compressive and splitting tensile strengths, with compressive strength increases of 24.01%, 12.62%, and 14.04% compared to AM2.0, AM1.5, and AM1.0, respectively, and splitting tensile strength increases of 85.10%, 46.95%, and 38.99% compared to AM2.0, AM1.5, and AM1.0, respectively.

As the curing age continues to increase, the strengths of specimens with different moduli show a certain degree of improvement. Comparatively, the AM1.2 specimen exhibits the highest compressive and splitting tensile strengths at different curing ages. After 28 days of standard curing, the compressive strengths of AM2.0~1.0 specimens are 48.96 MPa, 53.24 MPa, 61.68 MPa, and 52.11 MPa, respectively, while the splitting tensile strengths are 3.94 MPa, 3.86 MPa, 4.37 MPa, and 4.26 MPa, respectively. The compressive strength of the AM1.2 specimen is 25.98%, 15.85%, and 18.36% higher than those of AM2.0, AM1.5, and AM1.0, respectively, and the splitting tensile strength is 10.91%, 13.21%, and 2.58% higher than those of AM2.0, AM1.5, and AM1.0, respectively. From Figure 8c, it can be seen that at 1 day, the AM1.2 specimen has a significantly higher mix ratio compared to the other specimens. As the curing age increases, the growth rates of compressive strength in the AM1.2 and AM1.0 specimens are higher than those of splitting tensile strength, resulting in a certain degree of decrease in the tensile-to-compressive strength ratio. After 28 days of standard curing, the tensile-to-compressive strength ratios of the AM2.0 and AM1.5 specimens are slightly higher than those of AM1.2 and AM1.0.

Overall, a water glass modulus that is too high or too low negatively impacts the strength development of the cementitious material system. According to the experimental results, an activator modulus of 1.2 yields the most significant improvement in specimen strength, particularly in splitting tensile strength. This is because the modulus of water glass determines the polymerization state of silicon–oxygen tetrahedra. In high-modulus water glass solutions, the degree of polymerization is high, leading to a lower concentration of monomers and single polymers, which reduces reactivity compared to low-modulus water glass. Moreover, higher-modulus water glass exhibits greater viscosity, which hinders hydrolysis and delays the depolymerization and polycondensation processes in the “water glass–slag glass” system.

### 3.5. Four-Dimensional Evaluation

**Figure 9:**
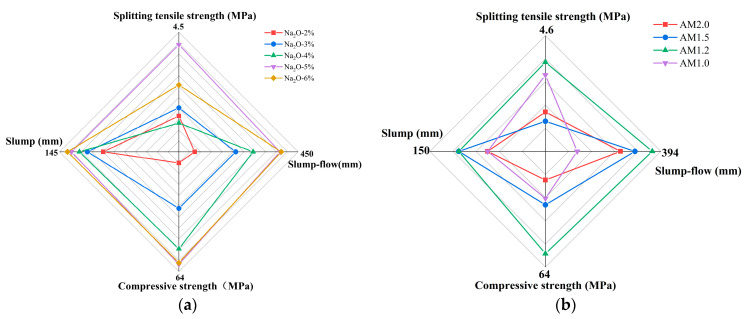
Four-dimensional evaluation diagrams for different alkali equivalents and moduli. (**a**) Alkali equivalents, (**b**) moduli.

Based on the experimental results, this study selected four indicators, compressive strength, splitting tensile strength, slump, and spread, and conducted a comparative analysis using a four-dimensional evaluation method [52]. Through multi-dimensional evaluation, the optimal parameters for alkali-activation control were proposed. The multi-dimensional evaluation of eco-friendly cementitious materials made from YRS under different alkali equivalents and activator moduli is shown in Figure 9. As can be seen from the figure, when the Na_2_O content is 5% and the activator modulus (AM) is 1.2, the workability and strength of the eco-friendly cementitious materials achieve a good balance. This is because an appropriate alkali equivalent and activator modulus can promote the rapid dissolution of raw materials, reduce the cohesion of the mixture paste, and improve the flowability of the mixture. On the other hand, the potential reactivity of slag is fully activated in a suitable alkaline environment, enabling the orderly development of matrix strength and avoiding issues such as insufficient strength growth due to under-activation or strength decline in later stages due to over-activation. This facilitates the smooth progression of the “depolymerization–polycondensation” reaction.

## 4. Influence Mechanism of Alkali Activator Characteristic Parameters on Characteristic Products and Microstructure

### 4.1. Characteristic Hydration Products

**Figure 10:**
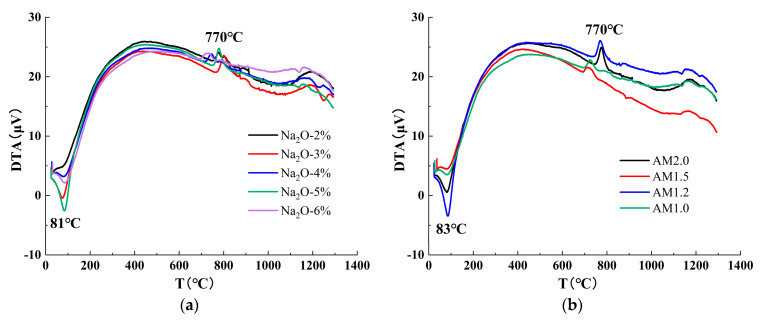
Alkali equivalent and modulus differential thermal analysis (DTA) curves. (**a**) Alkali equivalent, (**b**) modulus.

Figure 10 shows the results of a comprehensive thermal analysis for alkali-activated slag cement with a water glass modulus of 1.5 under standard curing at different ages. From both Figure 10a,b, it can be observed that the differential thermal analysis (DTA) curves for different alkali equivalents and activator moduli exhibit similar patterns, indicating that the alkali content and activator modulus do not alter the types of characteristic products. Additionally, two main thermal analysis peaks are identified in the curves, occurring in the temperature ranges of 60 °C to 100 °C and 700 °C to 800 °C, respectively.

The endothermic peak and mass loss observed in the range of 60 °C to 100 °C are primarily caused by the release of free and adsorbed water from the pore structure and gel-like hydration products (C-S-H and C-A-S-H) in the matrix. The exothermic peak and mass loss near 700 °C to 800 °C are due to the dehydration of the main reaction products, namely low calcium-to-silicon ratio C-S-H and C-A-S-H gels. Compared to traditional C-S-H, low calcium-to-silicon ratio C-S-H exhibits better thermal stability, while the incorporation of aluminum in C-A-S-H gels significantly enhances their thermal stability. Therefore, the thermal decomposition peaks of low calcium-to-silicon ratio C-S-H and C-A-S-H gels occur at higher temperatures.

No endothermic peak for Ca(OH)_2_ is observed around 450 °C, indicating that the products do not contain Ca(OH)_2_. The more prominent the characteristic peaks in the DTA curve, the greater the mass loss and the more intense the reaction. From Figure 10a, it can be seen that the mass loss of the two characteristic peaks first increases and then decreases with the increase in alkali equivalent, suggesting that selecting an appropriate alkali equivalent can significantly accelerate the reaction rate and effectively promote the formation of characteristic products. From Figure 10b, it is evident that the mass loss of the two characteristic peaks first increases and then decreases with the decrease in activator modulus, indicating that an appropriate activator modulus can significantly accelerate the reaction rate. Furthermore, compared to Figure 10a, the activator modulus has a more pronounced effect on the temperature range of 700 °C to 800 °C, indicating that while both the alkali equivalent and activator modulus can influence the reaction rate, the activator modulus can also affect the polymerization degree of the main hydration products. Therefore, selecting an appropriate activator modulus can promote the formation of silicon–oxygen tetrahedral groups with various polymerization degrees in the solution, effectively facilitating the transformation of C-A-S-H and C-S-H gels from monomers to polymers.

**Figure 11:**
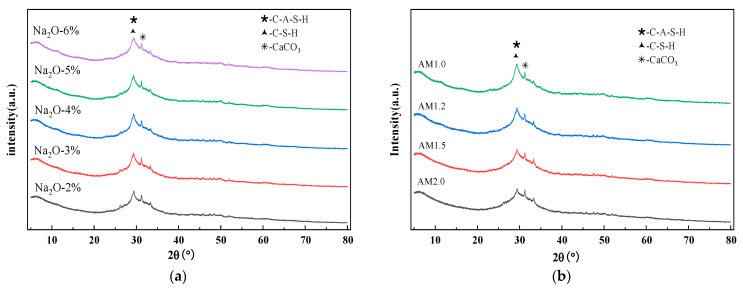
Comparative study of XRD patterns under different alkali equivalents and moduli. (**a**) Alkali equivalents, (**b**) modulus.

Figure 11a,b show the XRD patterns of matrix under different alkali equivalents and moduli, respectively. To avoid the influence of impurities and minerals in the sediment on the products, the sample preparation involved using a paste sample with the sediment removed. As can be seen from the figures, in both Figure 11a,b, the phases in the groups were similarly composed of amorphous gel. The peaks observed between the diffraction angles of 20°–40° are indicative of C-A-S-H, C-S-H, and CaCO_3_ in calcium-containing systems [53,54]. It can be found that the alkali equivalent and alkali modulus only affects the content of matrix products and does not cause the formation of new products. With the increase of alkali equivalent and alkali modulus, the peak intensities of C-(A)-S-H and CaCO_3_ increase.

### 4.2. Pore Structure

**Figure 12:**
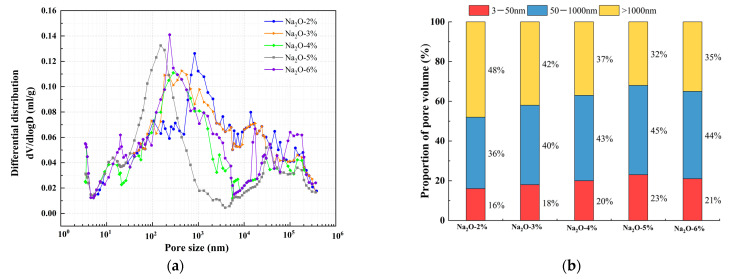
Comparative study of matrix pore characteristics under different alkali equivalents. (**a**) Relationship between dV/dlogD and pore size, (**b**) proportion of pore volume.

**Figure 13:**
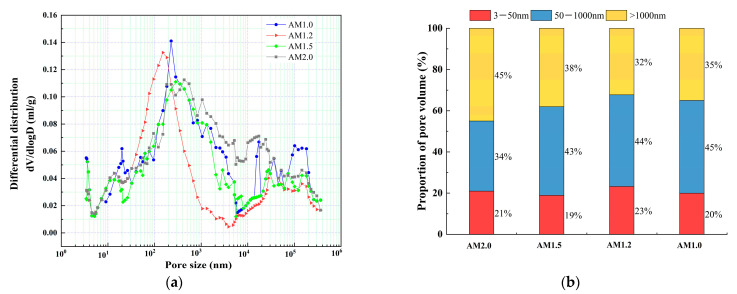
Comparative study of pore characteristics of matrix under different moduli. (**a**) Relationship between dV/dlogD and pore size, (**b**) proportion of pore volume.

The effect of alkali equivalent and activator modulus on pore structure at an age of 28 d was selected for MIP experiments, as shown in Figure 12 and Figure 13. According to the results of the mercury injection test, in order to better study the relationship between pore-volume distribution and strength, the pore size is divided into three intervals: 3–50 nm, 50–1000 nm, and >1000 nm. The pore structure has a significant effect on the strength development of the matrix, and the mechanisms of different sizes of pores have obvious differences. Gel pores (<50 nm) are mainly distributed inside C-S-H gel and between its particles. These pores have a positive effect on the densification of the matrix [55]. In the later curing process, gel pores provide a channel for water migration and promote the continuous hydration of incompletely reacted mineral admixtures (such as slag) to produce C-(A)-S-H gel and other products, so as to refine the pore structure and improve the compactness of the matrix, which is conducive to the stable development of strength [56]. In contrast, macropores larger than 1000 nm are generally considered to be harmful pores, and their adverse effects are mainly reflected in two aspects. First, macropores tend to become stress concentration points when subjected to stress. According to Griffith fracture theory, the larger the pore size, the lower the stress required for critical crack growth, which will significantly reduce the bearing capacity of the material. Second, these macropores often connect with each other to form a permeability network, and they are mainly concentrated in the aggregate slurry interface (ITZ region). The porosity of this region is high, and the existence of macropores will further weaken the interfacial bonding performance, making the material preferentially break from the ITZ region when stressed, which will seriously affect the overall mechanical properties of the material [57].

The relationship between dV/dlogD and the pore size of a matrix under different alkali equivalents is shown in Figure 12a. The pore volume of a matrix under different alkali equivalents is shown in Figure 12b. From the figure, it can be observed that when the alkali equivalent is within 5%, as the alkali equivalent increases, the most probable pore size significantly decreases, the proportion of large pores significantly decreases, and the proportion of gel pores smaller than 50 nm steadily increases. When the alkali content reaches 5%, the proportion of gel pores increases from 16% to 23%, while the proportion of large pores significantly decreases from 48% to 32%, and the most probable pore size decreases from 829 nm to 151 nm. This indicates that increasing the alkali equivalent promotes the progress of the “depolymerization–polycondensation” reaction, effectively refining the pore structure of the matrix, which is beneficial for strength development. This conclusion is consistent with the strength results. However, when the alkali equivalent continues to increase to 6%, the proportion of large pores increases instead of decreasing, rising from 32% to 35%, the proportion of gel pores decreases from 23% to 21%, and the most probable pore size increases from 151 nm to 237 nm. This is because an excessively high alkali equivalent causes the matrix to react too quickly, and the newly generated products occupy the active dissolution sites of the reactants, inhibiting further reaction and affecting the densification process of the matrix, resulting in a “coarsening” effect on the pore structure.

The relationship between dV/dlogD and the pore size of a matrix under different alkali moduli is shown in Figure 13a. The pore volume of a matrix under different alkali moduli is shown in Figure 13b. From the analysis of the figure, as the activator modulus decreases, the most probable pore size first decreases and then increases. When the activator modulus decreases from 2.0 to 1.2, the most probable pore size decreases from 433 nm to 151 nm. However, with a further decrease in the activator modulus, the most probable pore size increases from 151 nm to 227 nm. From Figure 13b, it can be seen that both excessively high and low activator moduli are unfavorable for the refinement of the pore structure. When the activator modulus decreases from 2.0 to 1.2, the proportion of gel pores increases from 21% to 23%, while the proportion of large pores significantly decreases from 45% to 32%. With a further decrease in the activator modulus, the proportion of large pores slightly increases from 32% to 35%, and the proportion of gel pores smaller than 50 nm decreases from 23% to 20%.

This is because, although the alkali content (Na_2_O) is relatively high and can promote the decomposition of the glass phase, a higher activator modulus leads to an increase in the polymerization degree of [SiO_4_]^4−^ itself, which is unfavorable for the depolymerization of the silicon–aluminum phase in the raw materials. This inhibits the polycondensation reaction process, ultimately resulting in a reduction of the main hydration product C-A-S-H in the cementitious system and slowing down the densification process of the matrix. On the other hand, an excessively low activator modulus causes the matrix to react too quickly, leading to the disorderly accumulation of randomly distributed C-A-S-H gels before they can properly transfer and arrange. This limits the growth and filling of the products, slowing down the refinement of the pore structure and also hindering the densification process of the matrix.

### 4.3. Matrix Microstructure

**Figure 14:**
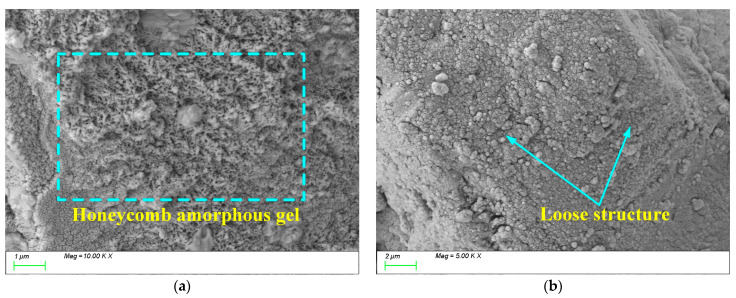
Microscopic images of 2%Na_2_O. (**a**) Characteristic product, (**b**) microstructure.

Figure 14 shows the SEM test results for 2% Na_2_O. From Figure 14a, it can be observed that when the alkali equivalent is low, the activation of slag powder is insufficient, and a large amount of unreacted slag powder remains within the matrix. Additionally, the low alkali equivalent leads to incomplete “depolymerization–polycondensation” reactions [58], resulting in insufficient growth of the main hydration products. Within the matrix, the C-S-H and C-A-S-H gels exhibit a honeycomb-like growth pattern with a low degree of polymerization, resulting in a relatively loose structure. This inadequate polymerization leads to the formation of numerous pores and microcracks within the matrix, as illustrated in Figure 14b. This observation aligns with the earlier strength analysis, indicating that the weak bonding and porous structure hinder the material’s overall mechanical performance and long-term durability.

**Figure 15:**
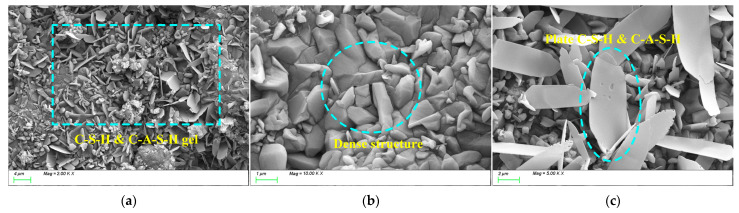
Microscopic image of 5%Na_2_O. (**a**) Characteristic product, (**b**) microstructure, (**c**) C-(A)-S-H gels composition characteristics.

When the alkali equivalent is increased to 5%, the SEM test results are as shown in Figure 15. From Figure 15a, it can be seen that compared to 2% Na_2_O, the 5% Na_2_O paste generates a large amount of wrinkled C-(A)-S-H gels, and the gel products grow sufficiently [59], effectively filling the voids in the matrix and forming a uniform, dense, continuous, and intact matrix, resulting in higher compressive strength. From Figure 15b,c, it can be observed that a large number of GGBFS particles have fully reacted, and the gel products are densely packed, fully grown, and exhibit a high degree of polymerization, leading to a dense matrix structure. This is because an appropriate alkali content can increase the dissolution rate of GGBS particles [60], thereby accelerating the reaction process and increasing the extent of the reaction, ultimately enhancing the generation of C-A-S-H and C-S-H.

**Figure 16:**
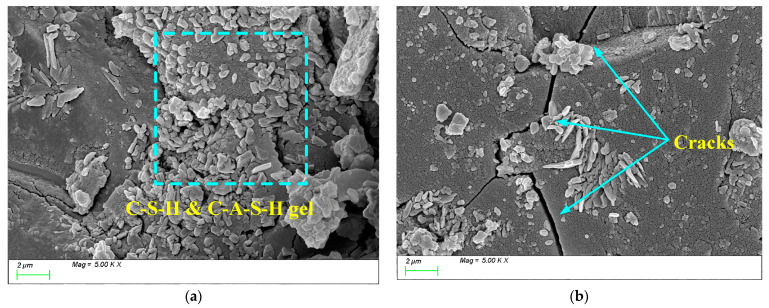
Microscopic images of 6%Na_2_O. (**a**) Characteristic product, (**b**) microstructure.

Figure 16 shows the SEM test results for 6% Na_2_O. From Figure 16a, it can be observed that when the alkali equivalent is further increased to 6%, a large amount of randomly distributed products are generated within the matrix, but they exhibit two characteristics: insufficient growth and uneven distribution. Additionally, from Figure 16b, it can be seen that cracks often form in areas where the products are concentrated. This is because an excessively high alkali equivalent accelerates the internal reaction within the matrix, causing hydration products to accumulate prematurely before they can properly transfer and integrate. This rapid accumulation coarsens the pore structure and induces microcracks at the aggregate–paste interface and within the paste itself. Consequently, the compactness of the matrix is reduced, negatively impacting the strength and long-term durability of the material.

**Figure 17:**
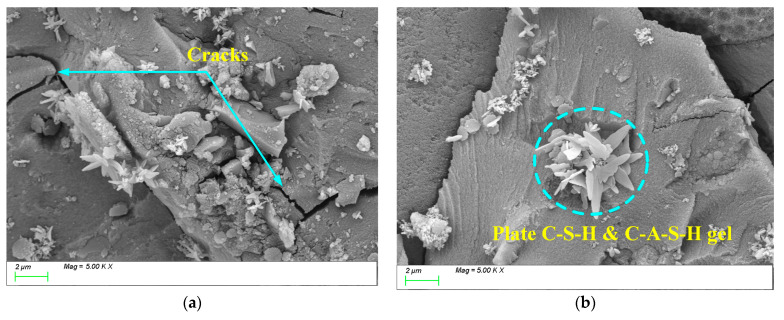
Microscope images of AM2.0. (**a**) Microstructure, (**b**) characteristic product.

Figure 17 shows the SEM test results for AM2.0. From Figure 17a, it can be seen that when the activator modulus is 2.0, the internal structure of the matrix is loose, and plate-like C-(A)-S-H gels are scattered on the surface of the matrix, with insufficient growth of the products. From Figure 17b, it can be observed that the overall structure of the matrix is loose and contains relatively more pore defects. This is because, in a high-modulus activation environment, the [SiO_4_]^4−^ in the water glass decreases, reducing the activation sites of the silicon–oxygen groups [61], resulting in insufficient growth of the products in the later stages and weak strength development. Additionally, some studies suggest that when the content of sodium silicate is high, excess silica may be released in the solution, precipitating in the form of amorphous silicate and forming a hardened body within the hardened paste [62]. This hardened body has low hardness and is unfavorable for strength development.

**Figure 18:**
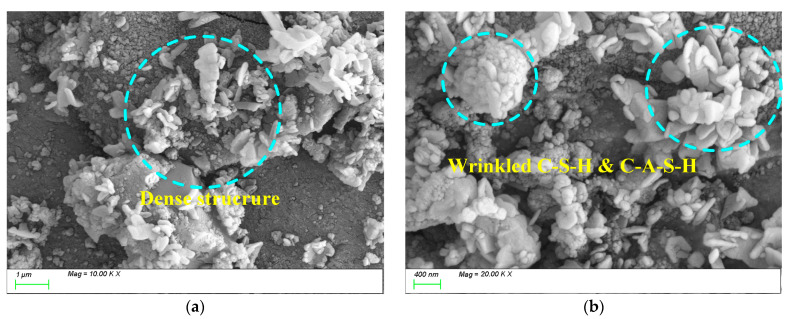
Microscope images of AM1.2. (**a**) Microstructure, (**b**) characteristic product.

Figure 18 shows the SEM test results for AM1.2. From Figure 18a,b, it can be observed that when the activator modulus is 1.2, a large amount of C-(A)-S-H and C-S-H hydration products are generated within the matrix, and they grow sufficiently, exhibiting a wrinkled and densely packed state. This is because, on the one hand, the alkali content of 5% effectively activates the reactivity of the slag powder, and on the other hand, an appropriate activator modulus promotes the growth of silicon–oxygen tetrahedral groups in the solution.

**Figure 19:**
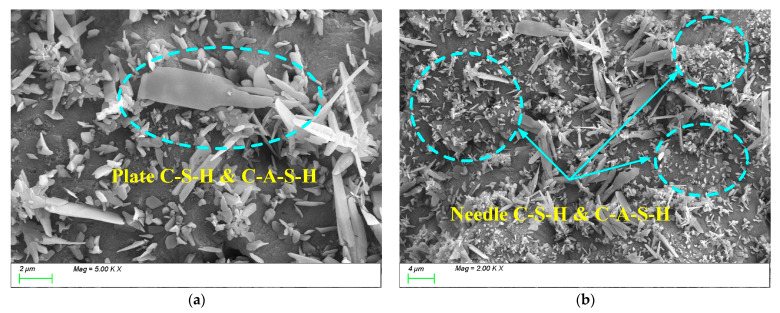
Microscope images of AM1.0. (**a**) Plate-like C-(A)-S-H, (**b**) needle-like C-(A)-S-H.

These C-(A)-S-H and low calcium-to-silicon ratio C-S-H gel particles interlock and connect, forming a dense three-dimensional network structure, effectively facilitating the transformation of C-A-S-H and C-S-H gels from monomers to polymers. As a result, the degree of polymerization of the gels in the products increases, and the compactness of the matrix improves. Figure 19 shows the SEM test results for AM1.0. From the figure, it can be seen that when the activator modulus is 1.0, a large amount of C-(A)-S-H and C-S-H hydration products are generated within the matrix, but the growth of the products is uneven, primarily exhibiting two growth states: plate-like (Figure 19a) and needle-like (Figure 19b). This is because, on the one hand, the low activator modulus effectively activates the reactivity of the slag powder in the early stages of the reaction, generating a large amount of plate-like gel products, as shown in Figure 19a. However, due to the excessively fast reaction, the surface is covered by a layer of hydration film, which acts as a physical barrier, slowing down the movement of ions to the particle surface and thus reducing the reaction with the particle elements. As a result, the later growth of C-(A)-S-H and C-S-H is insufficient, and the degree of polymerization is inadequate. Consequently, a large number of needle-like transitional gel products grow within the matrix in the later stages, slowing down the refinement of the pore structure and affecting the densification process, as shown in Figure 19b. This may be part of the reason why its later strength development is not as good as that of AM1.2.

## 5. Conclusions

This study investigated the effects of alkali equivalent and modulus on various critical properties of YRS-based ecological cementitious materials, including setting time, workability, compressive strength, splitting tensile strength, hydration heat, characteristic products, pore structure, and matrix microstructure. The key findings of the study are summarized as follows.

(1) Both the alkali equivalent and the activator modulus influence the alkali-activation reaction process, leading to a faster setting time for paste. Alkali modulus accelerates the setting time up to 1.2, after which it stabilizes. An increase in alkali equivalent shows a faster increase in setting time compared to the alkali modulus. When the alkali equivalent increases to more than 5%, the initial and final setting times significantly decrease, dropping from 101 and 110 min to 13 and 16 min, respectively. Further increases in the alkali equivalent result in minimal changes to the setting times.
(2) The workability of mixtures with different moduli is generally good. In comparison, the mixture with AM1.2 exhibits the best flowability. Compared to the activator modulus, the alkali equivalent has a more significant impact on the workability of the mixture. Increasing the alkali equivalent can effectively enhance the flowability of the mixture.
(3) Increasing the alkali equivalent and reducing the activator modulus can improve the hydration heat release rate and total hydration heat during the initial reaction period, shorten the induction period, and accelerate the entry into the acceleration period. After 72 h of cumulative heat release, the hydration heat of 3%~6% Na_2_O pastes increased by 16.83%, 100%, 203.22%, and 213.86%, respectively, compared to 2% Na_2_O. The hydration heat of AM1.0~1.5 increased by 20.74%, 18.65%, and 11.40%, respectively, compared to AM2.0.
(4) An appropriate alkali content and activator modulus can significantly accelerate the “dissolution–depolymerization–condensation” reaction process in the matrix, promoting rapid early strength development and stable long-term strength growth. An excessively high or low alkali content and activator modulus can lead to insufficient growth and uneven distribution of characteristic products, hindering the refinement of the matrix pore structure and resulting in weak strength development or even strength regression in later stages. Therefore, synergistic optimization and systematic control of the alkali equivalent and activator modulus are necessary.
(5) When the alkali equivalent and activator modulus are 5% and 1.2, respectively, the matrix exhibits excellent flowability, balanced and sustained hydration heat release, high early strength, and stable long-term strength growth. The 28 day compressive and splitting tensile strengths of the specimens can reach 61.68 MPa and 4.37 MPa, respectively.
(6) The determination of optimal alkali-activation parameters ensures steady progression of the “depolymerization–polycondensation” reaction and enables sustained strength development while preventing either insufficient reaction due to inadequate alkali activation or excessive pore coarsening and strength regression caused by over-activation. Although the current research focuses on alkali-activated slag Yellow River sediment composites due to experimental constraints, the parameter optimization methodology can be extended to the activation of other aluminosilicate solid wastes (such as red mud, metallurgical slag, steel slag, and tailings), providing a novel solution to the accumulation of aluminum industrial waste and facilitating the high-value utilization of solid waste resources.

## Figures and Tables

**Table 1:** Chemical compositions of YRS and GGBS (wt.%).

Minerals	SiO_2_	CaO	Al_2_O_3_	Fe_2_O_3_	K_2_O	TiO_2_	MgO	Other
YRS	68.64	8.40	12.33	3.25	2.55	0.74	2.05	2.04
GGBFS	32.47	41.06	14.52	0.28	0.44	1.25	7.08	2.9

**Table 2:** The chemical composition of sodium silicate.

SiO_2_/(%)	Na_2_O/(%)	H_2_O/(%)	Density/(g/cm^3^)	Modulus	Beaume
30	13.5	56.5	1.51	2.3	50

**Table 3:** Mix proportion for alkali activator characteristic parameters.

**No.**	**Sand**	**NaOH**	**SS**	**GGBFS**	**Water**
Na_2_O-2%	1.000	0.008	0.051	0.660	0.235
Na_2_O-3%	1.000	0.012	0.077	0.660	0.221
Na_2_O-4%	1.000	0.016	0.102	0.660	0.206
Na_2_O-5%	1.000	0.020	0.128	0.660	0.192
Na_2_O-6%	1.000	0.024	0.153	0.660	0.177
AM-2.0	1.000	0.006	0.208	0.646	0.140
AM-1.5	1.000	0.015	0.158	0.655	0.173
AM-1.2	1.000	0.020	0.128	0.660	0.192
AM-1.0	1.000	0.024	0.107	0.665	0.205

Na_2_O-2% means that the sodium oxide (Na_2_O) content in the activator system accounts for 2 wt.% of the slag, and the rest is analogized. AM-1.0 means that the activator (sodium silicate solution) has a SiO_2_/Na_2_O molar ratio of 1.0, and the rest is analogized.

**Table 4:** Grouping of the workability, mechanical, reaction progress, and microstructural property tests.

Properties	Performance Index	Specimen Size	Quantity
Setting time	initial setting time	—	—
	final setting time	—	—
Workability	slump	—	—
	slump–flow	—	—
Hydration heat	heat evolution rate	—	—
	accumulated hydration heat	—	—
Strength	compressive strength	100 mm × 100 mm × 100 mm	96
	splitting tensile strength	100 mm × 100 mm × 100 mm	96
Characteristic products	thermogravimetric analysis	40 mm × 40 mm × 40 mm	24
	X-ray diffraction analysis	40 mm × 40 mm × 40 mm	24
Microstructural properties	porosity	40 mm × 40 mm × 40 mm	24
	scanning electron microscopy	40 mm × 40 mm × 40 mm	24

## Data Availability

The original contributions presented in the study are included in the article, and further inquiries can be directed to the corresponding author.

## Nomenclature

YRS	Yellow River sediment	nm	Nanometer
C-S-H	Calcium silicate hydrate	P	Pressure
C-A-S-H	Calcium aluminum silicate hydrate	XRD	X-ray diffraction
AM	Activator modulus	m	Meter
MIP	Mercury intrusion porosimetry	SiO_2_	Silicon dioxide
AAM	Alkali-activated material	g	Gram
DTA	Differential thermal analysis	g.cm^−3^	Grams per cubic centimeter
EDS	Energy dispersive spectroscopy	${SO}_{4}^{2-}$	Sulfate ion
OPC	Ordinary Portland cement	N	Newton
AA	Alkali activator	SiO_2_	Silicon dioxide
AK	Alkali equivalent	SG	Specific gravity
mm	Millimeter	SEM	Scanning electron microscope
m^2^	Square meters	μW	Microwatt
m^2^·kg^−1^	Square meters per kilogram	NaOH	Sodium hydroxide
min	Minute	Vr	Pore diameter
MPa	Megapascal	%	Percentage
